# Investigating the relationship between monetary policy, macro-prudential policy and credit risk in Indonesia banking industry

**DOI:** 10.1016/j.heliyon.2023.e18229

**Published:** 2023-07-17

**Authors:** Cep Jandi Anwar, Indra Suhendra, Eka Purwanda, Agus Salim, Nur Annisa Rakhmawati, Ferry Jie

**Affiliations:** a Department of Economics, Faculty of Economics and Business, University of Sultan Ageng Tirtayasa, Banten, 42118, Indonesia; b STIE STEMBI, Bandung Business School, Bandung, Indonesia; c School of Business and Law, Edith Cowan University, Joondalup, Australia

**Keywords:** Monetary policy, Macro-prudential policy, Credit risk, Poolability tests, Pooled mean group estimation

## Abstract

Using a novel panel data set we study the influence of monetary and macro-prudential policies on non-performing loans as a measure of credit risk in Indonesian banking industry from Q1 2010 to Q4 2022. The panel homogeneity assumption was verified through the utilization of the Chow and Roy-Zellner tests. The findings showed that the model was not homogenous, necessitating the use of the Pooled Mean Group (PMG) estimator. The results indicated that monetary and macro-prudential policies significantly impacted credit risk. Furthermore, tight monetary and macro-prudential policies increased and reduced credit risk in the long run, respectively. The findings also showed that a loosening monetary policy reduced credit risk in the short run. Therefore, higher authorities must establish effective monetary and macro-prudential policies to reduce the non-performing loan ratio and maintain credit risk in Indonesia's banking industry.

## 1 Introduction

Monetary policy is critical to a central bank's macroeconomic stability in price maintenance and financial system protection [1,2]. A central bank influences the real economy by controlling the monetary policy rate and money supply. Subsequently, these policies impact financial markets for the banking sector, corporate bonds, and stocks [3]. Credit markets significantly impact the monetary policy transmission from the banking sector to the real economy [4]. Furthermore, borrowers may be vulnerable to changes in lending terms because the economy may be constrained by a rise in interest rates when balance sheets are fragile. The monetary policy's credit channel mechanism impacts the effectiveness of the financial markets and their capacity to meet the demands of borrowers and lenders [5,6]. Additionally, it influences the credit market rationing experienced by borrowers [7].

In this study, non-performing loans are utilised as a proxy for credit risk and may be used as the primary indicator in gauging credit risk that might damage the country's banking sector. Credit risk leads banks to lose existing loans as a result of bank credit failures; thus, each bank will establish credit management standards that include defining and executing processes that are compatible with bank risks, as well as monitoring the managed credit risk [8,9]. Aside from low credit quality, the negative consequences of NPL include rising operating expenses and decreased banking activity.

Monetary authority and market participants increasingly recognise systemic risk mitigation efforts alongside a financial crisis that can disrupt the economy [10]. As a financial authority with authority in macroprudential matters, Bank Indonesia (Central Bank of Indonesia) formulates macroprudential policies and conducts macroprudential supervision activities with the primary goal of mitigating systemic risk. It is believed that macroprudential policy complements with monetary policy to achieve price and financial stability.

Credit growth is actually normal due to financial consequences deepening in the economy. On the other hand, increasing credit, especially credit consumption can trigger aggregate demand above potential output and cause overheating. Due to banks' increased confidence in their clients' capacity to pay and their lack of care when extending credit to high-risk groups, there is a chance that these loans would default [11]. Long-term economic activity has a favorable impact on loan demand, whereas interest rates and inflation have a negative impact [12]. The ratio of non-performing loans to funds has an impact on the expansion of temporary short-term lending. Furthermore, there is a negative correlation between NPL and credit growth; the bigger the NPL, the less eager the banks are to offer credit.

The macro-prudential policy occupied a crucial status after the global financial crisis of 2008. In this case, the crisis catalysed the creation of macro-prudential regulation [13]. As a post-crisis solution, the macro-prudential policy aims to maintain financial system stability. This is an effort to reduce the possibility of systemic risks that disrupt the financial system. Macro-prudential policies emerged after the global economic crisis in 2008 as subprime mortgages by encouraging economic growth through lending. Therefore, these policies are useful for the central bank to monitor the soundness of the country's financial system [14].

In Indonesia, the use of monetary policy based on the inflation targeting framework has been successful in lowering inflation, promoting economic growth, and lowering interest rates [15]. However, these conditions have boosted the growth of excessive credit, which is the reason moral hazard for businesspeople has emerged. Market participants believe that the central bank has guaranteed all macroeconomic circumstances in order to promote growth activity towards the acquisition of risky assets at a larger profit. The increase in NPLs in the Indonesian banking industry was allegedly due to the influence of the real interest rate policy from Bank Indonesia. Bank Indonesia as an institution authorized to control interest rates has an effect on bank loans. High interest rates cause bank lending rates to rise as well so that these conditions can reduce the ability of debtors to repay loans. The result is an increase in the NPL ratio. According to Ref. [16] monetary policy can be influenced by internal factors of banks that extend credit and internal factors of parties receiving credit. Therefore monetary policy needs to be seen its effect on debtors and creditors.

The motivation of this study is an anomaly concerning the credit channel theory in the Indonesia case. It shows that the interest rates of the central bank are directly related to the supply of credits, and this is an anomaly between the real conditions and the existing theory [17]. The findings may be relevant to policymakers for two reasons, first, assessing overall asset quality and credit risk in the financial sector is a critical component of macroprudential monitoring. A solid knowledge of its causes allows the identification of important financial sector risks. Second, to provide standard scenarios for all financial institutions participating in such an exercise, regular stress tests of loan quality are increasingly based on macroeconomic assumptions. Loan quality stress testing was also an essential component of previously completed tests to rebuild trust in financial institutions.

In Indonesia, the credit situation appears to indicate monetary policy instrument imbalances, necessitating first determining the influence of these instruments and establishing the ideal monetary policy for the Central Bank of Indonesia to strive for when adopting its monetary policy stance. As a result, the purpose of this study is to investigate the Central Bank of Indonesia's current monetary policy mix to see if it has an effect on bank credit risk in Indonesia. There has been no prior research on the impact of monetary policy and macroprudential policy on credit risk using bank level data in Indonesia.

This study has highlighted and bridged the gaps existent in previous study, such as the absence of empirical evidence of the consequences of monetary policy and macroprudential policy on credit risk using bank level data in Indonesia, this research is expected to make many additions to the body of literature. There were no empirical evidence available to explain the association between monetary policy and macroprudential policy and credit risk using bank level data.

This study used non-performing loans as a proxy for credit risk. In line with this [18], stated that non-performing loans could be the main indicator in measuring the credit risk affecting a banking system [19]. found that credit risk causes banks to lose outstanding loans due to credit failures. Banks set standards by identifying and implementing procedures consistent with credit risks and measuring their management. This aims to prevent the deterioration of credit quality by increasing operational costs and worsening banking activities. In line with this, non-performing loans comprise the bad credit faced by banks or a condition where debtors cannot fulfil their obligations according to the provisions agreed upon [20]. A loosening monetary policy causes banks to increase credit interest rates and reduce borrowers’ ability to repay their debt, increasing non-performing loans.

Currently, there are a few studies have assessed the effect of macro-prudential policy on credit risk. The joint effect of monetary and macro-prudential policies on credit risk has also not been investigated. Therefore, this study aims to fill the gaps by examining the partial and joint effects of monetary and macro-prudential policies on credit risk. This approach differs from existing literature because it used three perspectives. First, the study used the Chow and Roy-Zellner tests to validate the homogeneous parameter hypothesis of pooled least squares estimation following the approach of [7]. Second, the Pooled Mean Group (PMG) estimator was applied to solve heterogeneity problems in the panel data. In line with this [21], found that PMG techniques capture the dynamism and variety of parameters. Third, the impact of macro-prudential and monetary policies on credit risk was robust. The study performed dynamic panel estimation and found a favorable correlation.

Section 2 of this study describes the constructs, methods, and models, while section 3 presents the data set used. Additionally, sections 4, 5 present the findings and recommendations for further studies.

## 2 Literature Review

According to Ref. [22], central bankers concurred that the distress of the financial sector during financial crises was primarily attributable to banks' credit risk, as indicated primarily by banks' non-performing loan levels. This is similar to the arguments of [23] financial instability hypothesis. Central bank policy rate influences credit risk because it affects the interest rate. According to, the central bank policy rate significantly impacts the banking sector. A decrease in interest rates accelerates the reduction in commercial bank lending rates and contributes more to economic development. This finding supports [5,[24], [25], [26], [27]] that the central bank policy rate positively affects non-performing loans. An increase in policy interest rates raises the interest rates on bank loans charged to the public, increasing bank risks. However [4,28,29], found that policy interest rates negatively affect non-performing loans. The studies stated that credit risk is significantly affected when banks encourage lending to the public at low-interest rates.

Previous studies have examined the impact of monetary and macro-prudential policies on the banking sector [30]. found that the policies could reduce credit movements. Furthermore [4,28,29], showed that monetary easing increases bank risks [5]. also stated that changes in monetary policy transmission affect non-performing loans and result in macroeconomic changes [29]. found that macro-prudential policies could suppress pro-cyclical measures due to credit growth. Moreover [5,24,26], found a positive relationship between monetary policy and credit risk. Therefore, central banks need to implement monetary and macro-prudential policies simultaneously. This would accelerate the objective of transmitting monetary policy and creating a stable financial system that withstands shocks caused by credit failures. Studies on the application of banking policies simultaneously in regulating credit risk have mostly analyzed the effect of only one policy.

The effect of macro-prudential policy on the banking sector was analyzed by Refs. [13,14,19,[30], [31], [32]]. [31] found that the policy was effective in stabilizing the banking system in Nigeria. Furthermore [13,14,19,30,32], showed that macro-prudential policy significantly as well as positively impacts financial stability.

Credit risk is also influenced by inflation, which is a continuous increase in the general price level [33,34]. stated that inflation negatively affects non-performing loans. An increase in inflation forces the central bank to implement a tight monetary policy by raising the interest rate, reducing the credit risk. In contrast [29], showed that inflation positively affects non-performing loans. The study stated that higher inflation without an increase in profits earned by debtors weakens the ability to pay debts, increasing the credit risk.

[[35], [36], [37], [38]] showed that GDP growth negatively affects non-performing loans. The relationship between non-performing loans and GDP growth occurs in economic activity. In this case, a decline in GDP is a common characteristic of a recession that causes an economic downturn. This condition is characterized by a drastic drop in people's income and consumption, increasing credit risk and reducing bank profits.

The moral hazard by the borrower to pay off the debt on the specified date often increases credit risk problems. According to Ref. [34], moral hazard is the risk that one party in a transaction act in a way detrimental to the other party. In this regard, banks need to include terms or agreements in the loan contract. The agreements and statements relate to the bank's expectations regarding the borrower's behavior. This could be implemented to combat the moral hazard after the loan is made. Moral hazard is the behavior of shareholders, management, or banking debtors that violate corporate ethics and applicable law with hidden intentions or actions for their benefit [39].

Other factors contributing to internal banking credit risk are Loan Deposit Ratio (LDR) and Capital Adequacy Ratio (CAR) as bank liquidity and capital, respectively [39]. showed that bank liquidity negatively impacts non-performing loans for commercial banks in MENA countries [26,35,40]. found that bank liquidity reduced non-performing loans. However [41], showed that bank liquidity positively impacts non-performing loans [[42], [43], [44], [45]]. found that a higher capital ratio reduced bank credit risk.

The following hypotheses will be raised based on the analysis of panel data.

H1 Central bank rate has a positive and significant effect on the NPLs.

H2 Macroprudential policy has a negative and significant effect on the NPLs.

H3 Inflation has a negative and significant effect on the NPLs.

H4 Economic Growth has a positive and significant effect on the NPLs.

H5 Loan to deposit ratio has a positive and significant effect on the NPLs.

H6 Capital adequacy ratio has a positive and significant effect on the NPLs.

## 3 Data and Methodology

### 3.1 Data

The dependent variable was non-performing loans as a proxy of credit risk. Data on non-performing loans were taken from Indonesian banking reports. Furthermore, the first independent variable was Indonesia's central bank policy rate as a measure of monetary policy. The data on this variable were obtained from Bank Indonesia. The second independent variable was macro-prudential policy, whose data were taken from Ref. [46]. Data on the control variable of Inflation and GDP growth were sourced from the world economic indicator by World Bank. Additionally, data on LDR and CAR were taken from Indonesian banking reports. This study used quarterly data from Q1 of 2010 to Q4 of 2022 on 42 commercial banks registered on Indonesia Stock Exchange. Variable definitions show in Table 1.

**Table 1:** Variable definitions.

Variable Name	Abbreviation	Type	Measurement	Sources	Unit of Measurement
Non-Performing Loans	NPL	Dependent	The ratio of unpaid credit to total credit as a proxy for credit risk	Indonesian Banking Reports	Percent
Central Bank Rate	CB Rate	Independent	Central bank interest rate as monetary policy stand	Bank Indonesia	Percent
Macroprudential Policy Index	Mapp	Independent	A weighted index of 12 global macroprudential indicators	[56]	Index
Inflation	INF	Independent	The change in the current consumer price index	The World Bank (World Development Indicator)	Percent
Economic Growth	EG	Independent	An increase in the production of economic goods and services in one period of time compared with a previous period	The World Bank (World Development Indicator)	Percent
Loan to Deposit Ratio	LDR	Independent	A ratio of banks' total loans to total deposits	Indonesian Banking Reports	Percent
Capital Adequacy Ratio	CAR	Independent	A ratio of bank capital and risk-weighted assets	Indonesian Banking Reports	Percent

Table 2 illustrates the descriptive statistic of the data. Regarding the NPL, it can be seen that the minimum is 0.6152% and the highest is 22.27%. Central bank rate has a minimum value of 4.25% occurred in 2017 Q3 and a maximum value of 62.79% occurred in 1998 Q1-1998 Q4, during the economic and financial crisis. An additional fact relates to the macroprudential policy, the average is 0.2639, with a maximum of 0.3948 and a minimum of 0.0833. In terms of inflation, it can be noticed that although the average inflation achieved by Indonesia is relatively high (4.4226%). The maximum inflation was 8.61% and the country with the minimum inflation was 1.37.

**Table 2:** Descriptive statistic.

Variable	Mean	Std Dev.	Min.	Max.
NPL	2.9701	2.2875	0.6152	22.2700
CB Rate	5.5903	1.3316	3.500	7.7500
Mapp	0.2639	0.1009	0.0833	0.3948
INF	4.4226	1.9212	1.3700	8.6100
EG	4.6564	1.8361	−2.0700	6.2075
LDR	85.1745	25.9751	44.9934	223.8519
CAR	24.1750	10.0540	13.1073	55.5942

Concerning economic growth, the average economic growth in Indonesia during the study period was 4.6564, with a minimum value of −2.07 and a maximum of 6,20. For bank specific data, the first is LDR. The average of LDR is 85.1745, with a maximum of 223.8519 and a minimum of 44.9934. Meanwhile, CAR data shows that the average in banking in Indonesia is 24.1750%. The highest ratio of CAR was 55.5942% and the lowest was 13.1073%.

Table 3 reveals the correlation matrix between variables. Central bank rate has a negative correlation with macroprudential policy and CAR with coefficients of −0.6463 and −0.2276. This value indicates that there is a negative correlation between central bank rate and macroprudential policy at 65%, and central bank rate and CAR at 23%. However, the central bank rate has a positive correlation with inflation, economic growth and LDR with coefficients of 0.6753 (67%), 0.4199 (42%), and 0.0211 (2%). Macroprudential policy has a negative correlation with inflation (−0.5027), and economic growth (−0.4148). Meanwhile, macroprudential policy has a positive correlation with LDR (0.0692), and CAR (0.2291). Inflation has a positive correlation with economic growth (0.5295), and LDR (0.2160) but has a negative correlation with CAR (−0.1368). Economic growth has a negative correlation with LDR (−0.0004), and CAR (−0.1016). Finally, LDR has a positive correlation with CAR with coefficient of 0.4043 (40%).

**Table 3:** Correlation matrix.

Variable	CB Rate	Mapp	INF	EG	LDR	CAR
CB Rate	1.0000					
Mapp	−0.6436***	1.0000				
INF	(0.0000) 0.6753***	−0.5027***	1.0000			
EG	(0.0000) 0.4199***	(0.0000) 0.4148***	0.5295***	1.0000		
LDR	(0.0000) 0.0211	(0.0000) 0.0692***	(0.0000) 0.0264	−0.0004	1.0000	
CAR	(0.3236) 0.2276*** (0.0000)	(0.0012) 0.2291*** (0.0000)	(0.2160) 0.1368*** (0.0000)	(0.9822) 0.1016*** (0.0000)	0.4043*** (0.0000)	1.0000

Notes: *** denotes significance at the 1% level.

### 3.2 Econometrics methodology

The study models employed an econometric technique known as panel data regression. Macroeconomic variables of inflation and economic growth as well as bank microdata such as LDR and CAR were incorporated when assessing the effects of monetary as well as macro-prudential policies on non-performing loans. The models employed are as follows:

(1) $${NPL}_{it}=\beta_{0}+{\beta_{1}CBRate}_{it}+{\beta_{2}\;\mathrm{INF}}_{it}+{\beta_{3}Growth}_{it}+{\beta_{4}LDR}_{it}+{\beta_{5}CAR}_{it}+\varepsilon_{it}$$

(2) $${NPL}_{it}=\beta_{0}+{\beta_{1}MAPP}_{it}+{\beta_{2}\;\mathrm{INF}}_{it}+{\beta_{3}Growth}_{it}+{\beta_{4}LDR}_{it}+{\beta_{5}CAR}_{it}+\varepsilon_{it}$$

(3) $${NPL}_{it}=\beta_{0}+{\beta_{1}IR}_{it}+{\beta_{2}MAPP}_{it}+{\beta_{3}\;\mathrm{INF}}_{it}+{\beta_{4}Growth}_{it}+{\beta_{5}LDR}_{it}+{\beta_{6}CAR}_{it}+\varepsilon_{it}$$

This study built three different models. In models 1 and 2, the study investigated the partial effects of monetary and macro-prudential policies on credit risk. Model 3 examined the combined effect of the two policies on credit risk. The study added some control variables, including inflation, economic growth, LDR, and CAR. It referred to Refs. [28,29,33,35,45] that inflation determines credit risk. Economic growth was included following [4,5]. Furthermore, bank-specific variables such as LDR were used by referring to Refs. [26,35,39,40]. The study also added CAR based on [[42], [43], [44], [45]].

The poolability test was used to determine the variation of the equation's parameter varied between nations. A pooled least squares model represented a behavioral equation with similar parameters in time and across groups. The unconstrained model exhibited the same behavior but with varied parameters over time and between groups [47]. The unrestricted model for each cross-section was in Eq (4) as follows:

(4) $$y_{i}=Z_{i}\delta_{i}+u_{i}\;i=1,2,\ldots .,N$$

where $y_{i}^{\prime }=\left(y_{i1},\ldots ,y_{iT}\right),Z_{i}=\left[iT,X_{i}\right]$ and $X_{i}\;is\;T\;x\;K$. $\delta_{i}^{\prime }\;is\;1\;x\;\left(K+1\right)\text{,}$ and $u_{i}$ is Tx1. $\delta_{t}^{\prime }$ is the change depending on the specific equation being used. The constrained model is described in Eq (5) as follows:

(5) y=Zδ+u

where $Z^{\prime }=\left(Z_{1}^{\prime },Z_{2}^{\prime },\ldots ,Z_{N}^{\prime },\right),u^{\prime }=\left(u_{1}^{\prime },u_{2}^{\prime },\ldots ,Z_{u}^{\prime },\right)$.

The poolability test's null hypothesis in Eq (6) was:

(6) $$H_{0}:\delta_{i}=\delta \;against\;H_{1}:\delta_{i}\neq \delta$$

The pooling least squares estimator was not poolable because the assumption of homogeneity was not held. Therefore, the study used the Mean Group estimation (MG), Pool Mean Group Estimation (PMG) and Dynamic Fixed Effect (DFE) estimation. We run the Hausman test to check MG vs PMG and PMG vs DFE to select the best estimation [21]. created the PMG estimation using an Autoregressive Distributive Lag (ARDL) structure and estimated the ARDL approach as an Error Correction Model. This estimation covered the long and short-run effects. Every cross-section may have intercepts, short-run coefficients, and error variances allowed by the PMG estimator. However, long-run coefficients are always subject to the same restriction. The PMG model was based on the ARDL approach (*p,q,q, …,q*) is in Eq (7).

(7) $$y_{it}=\sum_{j=1}^{p}\lambda_{ij}y_{i,t-1}+\sum_{j=0}^{q}\delta_{ij}^{\prime }x_{i,t-j}+\mu_{i}+\varepsilon_{it}$$

Following [36], a dynamic heterogeneous panel was estimated based on the ARDL approach (*p*_*i*_*, q*_*i*_*, k*_*i*_*, l*_*i*_*, m*_*i*_) model in Eq (8) as follows:

(8) $${\Delta y}_{it}=\varphi_{i}\;y_{i,t-1}+x_{i}\beta_{i}+\sum_{j=1}^{p-1}\lambda_{ij}^{*}\;\Delta \;y_{i,t-j}+\sum_{j=1}^{q-1}\delta_{ij}^{*}\;\Delta \;x_{i,t-j}+\mu_{i}+\varepsilon_{it}$$

hence, Eq (1) becomes Eq (9):

(9) $${NPL}_{it}=\alpha_{i}+\sum_{j=1}^{p_{i}}\beta_{ij}{NPL}_{i,t-1}+\sum_{j=0}^{q_{i}}\delta_{ij}{CB\;Rate}_{i,t-j}+\sum_{j=0}^{k_{i}}\theta_{ij}{\mathrm{INF}}_{i,t-j}+\sum_{j=0}^{l_{i}}\varphi_{ij}{Growth}_{i,t-j}+\sum_{j=0}^{m_{i}}\partial_{ij}{LDR}_{i,t-j}+\sum_{j=0}^{n_{i}}\varphi_{ij}{CAR}_{i,t-j}+\varepsilon_{it}$$

(10) $${\Delta NPL}_{it}=\varphi_{i}\;\left({CG}_{i,t-1}-\alpha_{i}^{*}-\delta_{i}^{*}\;{CB\;Rate}_{it}+\theta_{i}^{*}\;{\mathrm{INF}}_{it}+\varphi_{i}^{*}{Growth}_{it}++\partial_{i}^{*}{LDR}_{it}++\varphi_{i}^{*}{CAR}_{it}\right)+\sum_{j=1}^{p_{i-1}}\beta_{ij}^{**}{\Delta NPL}_{i,t-j}+\sum_{j=0}^{q_{i-1}}\delta_{ij}^{**}\Delta {CB\;Rate}_{i,t-j}+\sum_{j=0}^{k_{i-1}}\theta_{ij}^{**}\Delta {\mathrm{INF}}_{i,t-j}+\sum_{j=0}^{l_{i-1}}\theta_{ij}^{**}\Delta {Growth}_{i,t-j}+\sum_{j=0}^{m_{i-1}}\theta_{ij}^{**}\Delta {LDR}_{i,t-j}+\sum_{j=0}^{n_{i-1}}\theta_{ij}^{**}\Delta {CAR}_{i,t-j}+\varepsilon_{it}$$

where Eqs (10), (11) are the long and short-run PMG estimator equations, respectively.

Dynamic panel data was used to show that the effect of monetary and macro-prudential policies on credit risk was robust. The study also used [48] First Difference GMM (FD-GMM) estimation to account for potential endogeneity.

(11) $$Y_{i,t}-Y_{i,t-1}=\gamma \;\left(Y_{i,t-1}-Y_{i,t-2}\right)+\beta \left(X_{i,t}-X_{i,t-1}\right)+\left(\varepsilon_{i,t}-\varepsilon_{i,t-1}\right)$$

## 4 Empirical results

### 4.1 Pooled least squares estimation

First, the 12 models were estimated by running POLS estimation in Table 4. The results showed that the central bank rate significantly and negatively affected non-performing loans in model 1 but not in model 3. Meanwhile, the macro-prudential policy significantly and positively influenced non-performing loans. The findings also showed that inflation significantly and negatively affected non-performing loans in all models. Economic growth significantly and negatively affected non-performing loans at a 1% significance level in models 1 and 3 b. Meanwhile, the estimation results for models 2, 3c, and 3 d showed a significant negative effect at the 10% significance level. LDR insignificantly and positively impacted non-performing loans. Additionally, CAR showed a significant positive effect on non-performing loans in the three models.

**Table 4:** Pooled least squares estimation.

Variable	Model 1a	Model 1 b	Model 1c	Model 1 d	Model 2a	Model 2 b	Model 2c	Model 2 d	Model 3a	Model 3 b	Model 3c	Model 3 d
CB Rate	−0.2351*** (0.0484)	−0.2215*** (0.0486)	−0.2218*** (0.0486)	−0.2363*** (0.0496)					−0.0673 (0.0546)	−0.0670 (0.0545)	−0.0680 (0.0546)	−0.0834 (0.0552)
MAPP					4.3190*** (0.5400)	4.1272*** (0.5512)	4.1133*** (0.5537)	4.2654*** (0.5607)	3.9576*** (0.6143)	3.7680*** (0.6239)	3.7447*** (0.6278)	3.8338*** (0.6292)
INF	−0.1464*** (0.0335)	−0.1092*** (0.0360)	−0.1100*** (0.0360)	−0.1089*** (0.0360)	−0.1424*** (0.0283)	−0.1206*** (0.0310)	−0.1211*** (0.0311)	−0.1240*** (0.0311)	−0.1204*** (0.0335)	−0.0988*** (0.0357)	−0.0991*** (0.0358)	0.0974*** (0.0538)
EG		−0.0860*** (0.0306)	−0.0854*** (0.0306)	−0.0859*** (0.0306)		−0.0530* (0.0308)	−0.0530* (0.0309)	−0.0529* (0.0308)		−0.0529*** (0.0308)	−0.0529* (0.0308)	−0.0527* (0.0308)
LDR			0.0010 (0.0010)	0.0017 (0.0011)			0.0002 (0.0010)	0.0010 (0.0011)			0.0003 (0.0010)	0.0012 (0.0011)
CAR				−0.0040 (0.0028)				−0.0047* (0.0027)				−0.0053* (0.0028)
Constant	4.9325*** (0.2081)	5.0927*** (0.2155)	5.0074*** (0.2321)	5.1248*** (0.2460)	2.4601*** (0.2372)	2.6614*** (0.2645)	2.6438*** (0.2725)	2.6640*** (0.2727)	2.8348*** (0.3854)	3.0338*** (0.4024)	3.0174*** (0.4054)	3.1245*** (0.4091)
No. Of Cross-sections	42	42	42	42	42	42	42	42	42	42	42	42

The results showed that the effects of the central bank rate and macro-prudential policy on credit risk were significant but contrary to expectations. These findings implied that central bank rate and macro-prudential policy wrongly explained the credit risk in commercial banks in Indonesia, assuming a homogeneity coefficient between countries [7]. stated that the insignificant effect of the explanatory variables on the dependent variables might be caused by inappropriate econometric estimation. Therefore, the assumption of the POLS estimation needed to be checked.

### 4.2 Poolability tests

This study conducted a poolability test under the presumptions of homoskedasticity and normally distributed errors as presented in Table 5. First, the chow test was performed on the four models. The results showed that the probability for each group is 0.000, rejecting the null hypothesis. Second, the Roy-Zellner test for Equations (1)–(3) was conducted, with a p-value of 0.0000 for each test. The findings implied that the hypothesis of slope homogeneity was rejected. Therefore, the panel data could not be pooled in terms of the cross-section.

**Table 5:** Poolability test.

Variable	Model 1a	Model 1 b	Model 1c	Model 1 d	Model 2a	Model 2 b	Model 2c	Model 2	Model 3a	Model 3 b	Model 3c	Model 3 d
Chow Test	16.16***	11.81***	13.43	14.71***	26.96***	19.31***	17.92	19.92***	21.30***	17.20***	16.35***	18.45***
	(0.0000)	(0.0000)	(0.0000)	(0.0000)	(0.0000)	(0.0000)	(0.0000)	(0.0000)	(0.0000)	(0.0000)	(0.0000)	(0.0000)
	[82, 2099]	[123, 2057]	[164, 2015]	[205,1973]	[82, 2099]	[123, 2057]	[164, 2015]	[205,1973]	[123,2057]	[164, 2015]	[205, 1973]	[246,1931]
Roy-Zellner Test	1324,96	1453,02	2203.08	3016.08***	2210.42	2374.83	2939.23	4084.47***	2620.02	2820.93	3350.80	4538.84***
	(0.0000)	(0.0000)	(0.0000)	(0.0000)	(0.0000)	(0.0000)	(0.0000)	(0.0000)	(0.0000)	(0.0000)	(0.0000)	(0.0000)
	[82]	[123]	[164]	[205]	[82]	[123]	[164]	[205]	[123]	[164]	[205]	[246]
No of Cross-Sections	42	42	42	42	42	42	42	42	42	42	42	42
No of Observations	2184	2184	2184	2184	2184	2184	2184	2184	2184	2184	2184	2184

Notes: *** denotes significance at the 1% level. Figures in parentheses are p-value.

### 4.3 estimation

We then run the MG (Table 8), PMG (Table 6), and DFE (Table 9) estimations. To select the best estimation, we apply the hausman test, MG vs PMG (Table 10) and PMG vs DFE (Table 11), and find that PMG is the best estimation. The PMG estimation was performed to solve the heterogeneity problem in the panel data. The results in Table 6 show that the Akaike Info Criterion (AIC) was used to select the best lag lengths for each variable in the PMG estimate. The optimal lag of the ARDL model indicated 2, 1, 1, 1, and 1. Moreover, the Error Correction Term (ECT) was significant and negative. This indicated that any short-run deviation would move back to the long-run equilibrium path. The negative and significant coefficient denoted that the explanatory factors in the model converged when the non-performing loans were subject to a shock and adjusted to the long-run equilibrium. The findings also showed that the short-run disequilibrium would be corrected quarterly by 6.06%–18.55%. Additionally, the PMG estimator reached a long-run equilibrium in about a year.

**Table 6:** PMG estimation.

Variable	Model 1a	Model 1 b	Model 1c	Model 1 d	Model 2a	Model 2 b	Model 2c	Model 2 d	Model 3a	Model 3 b	Model 3c	Model 3 d
**Long-run coefficients**												
CB Rate	0.7479*** (0.0982)	0.2731** (0.1290)	0.4357*** (0.0489)	0.3436*** (0.0421)					0.0635*** (0.0295)	0.0948*** (0.0297)	0.2777*** (0.0388)	0.2291*** (0.0267)
MAPP					−2.9957*** (0.3996)	−4.8512** (2.4661)	−2.0125** (0.9322)	−4.3877*** (1.0678)	−1.0692*** (0.4026)	−1.3482*** (0.3778)	−1.6139** (0.8077)	−2.8290*** (0.8908)
INF	−0.3197*** (0.1156)	−0.6407*** (0.1041)	−0.2542*** (0.0359)	−0.1847*** (0.0302)	−0.0215*** (0.0061)	−0.0737 (0.0656)	0.0050 (0.0170)	0.2689*** (0.0299)	−0.0858*** (0.0280)	−0.1161*** (0.0286)	−0.0397 (0.0274)	−0.1013*** (0.0222)
EG		0.4824*** (0.0736)	−0.0726*** (0.0231)	−0.0898*** (0.0178)		0.6361*** (0.0783)	0.0628** (0.0289)	−0.0375 (0.0286)		−0.0799*** (0.0222)	0.1697*** (0.0256)	0.0082 (0.0278)
LDR			−0.0034*** (0.0007)	−0.0027*** (0.0008)			−0.0016 (0.0015)	−0.0009** (0.0004)			0.0035*** (0.0007)	0.0037*** (0.0004)
CAR				−0.0274*** (0.0037)				−0.0066* (0.0036)				−0.0201*** (0.0032)
**Short-run coefficients**												
ECTt-1	−0.0737*** (0.0133)	−0.0861*** (0.0157)	−0.0802*** (0.0193)	−0.0606*** (0.0214)	−0.1534*** (0.0156)	−0.1265*** (0.0338)	−0.1077*** (0.0117)	−0.0730*** (0.0273)	−0.1863*** (0.0256)	−0.1399*** (0.0267)	−0.1855*** (0.0356)	−0.1586*** (0.0473)
Δ(NPL (-1))	0.3457*** (0.0467)	0.3249*** (0.0479)	0.5011*** (0.0424)	0.5537*** (0.0491)	0.6873*** (0.0277)	0.3648*** (0.0421)	0.7276*** (0.0242)	0.0055 (0.0046)	0.3309*** (0.0453)	0.3399*** (0.0490)	0.6080*** (0.0382)	0.6090*** (0.0045)
Δ(CB Rate)	−0.1171*** (0.0029)	−0.1123** (0.0474)	−0.0607** (0.0278)	−0.0331 (0.0225)					−0.1177*** (0.0331)	−0.0499* (0.0266)	−0.0917 (0.0656)	−0.0599 (0.0589)
Δ(MAPP)					0.3066 (0.6263)	1.9909 (2.4016)	3.2493*** (1.3246)	0.9134 (1.3298)	−0.5676 (1.1246)	3.8894*** (1.2017)	2.6616 (2.3171)	2.5642 (2.4156)
Δ(INF)	0.0179 (0.0149)	0.0521** (0.0212)	0.0142 (0.0144)	0.0086 (0.0115)	−0.0140* (0.0072)	0.0077 (0.0174)	−0.0034 (0.0067)	−0.0207* (0.0122)	−0.0113 (0.0134)	0.0054 (0.0126)	−0.0036 (0.0297)	0.0026 (0.0282)
Δ(EG)		−0.0208 (0.0176)	0.0093 (0.0281)	0.0045 (0.0214)		−0.0742*** (0.0259)	0.0072 (0.0245)	0.0125 (0.0240)		−0.0299 (0.0271)	−0.0334 (0.0263)	0.0050 (0.0213)
Δ(LDR)			0.0304 (0.0196)	0.0174 (0.0171)			0.0130 (0.0143)	0.0097 (0.0186)			0.0290 (0.0184)	0.0111 (0.0158)
Δ(CAR)				0.0286 (0.0312)				0.0272 (0.0380)				0.0188 (0.0418)

Notes: *** and ** denote the significance at the 1% and 5% levels, respectively. Figures in parentheses are standard errors.

**Table 7:** Panel GMM estimation (full sample).

Variable	Model 1a	Model 1 b	Model 1c	Model 1 d	Model 2a	Model 2 b	Model 2c	Model 2 d	Model 3a	Model 3 b	Model 3c	Model 3 d
NPL (−1)	0.7242*** (0.0004)	0.7223*** (0.0005)	0.7208*** (0.0006)	0.7244*** (0.0007)	0.6931*** (0.0004)	0.6913*** (0.0004)	0.6993*** (0.0006)	0.7067*** (0.0006)	0.7072*** (0.0006)	0.7044*** (0.0005)	0.7092*** (0.0006)	0.7175*** (0.0007)
CB RATE	0.0785*** (0.0007)	0.0690*** (0.0010)	0.0554*** (0.0022)	0.0879*** (0.0026)					0.0833*** (0.0011)	0.0768*** (0.0013)	0.0572*** (0.0022)	0.0854*** (0.0027)
MAPP					−0.7230*** (0.0146)	−0.5203*** (0.0284)	−0.6848*** (0.0782)	−0.7958*** (0.0936)	−0.2529*** (0.0311)	−0.0736*** (0.0233)	−0.3339*** (0.0962)	−0.4393*** (0.0519)
INF	−0.0074*** (0.0001)	−0.0257*** (0.0005)	−0.0292*** (0.0012)	−0.0381*** (0.0013)	−0.0105*** (0.0002)	−0.0092*** (0.0001)	−0.0191*** (0.0006)	−0.0195*** (0.0005)	−0.0132*** (0.0002)	−0.0313*** (0.0005)	−0.0346*** (0.0014)	−0.4271*** (0.0014)
EG		0.0567*** (0.0007)	0.0515*** (0.0016)	0.0485*** (0.0012)		0.0622*** (0.0009)	0.0569*** (0.0014)	0.0533*** (0.0012)			0.0539*** (0.0017)	0.0502*** (0.0013)
LDR			0.0072*** (0.0004)	0.0045*** (0.0004)			0.0081*** (0.0003)	0.0063*** (0.0003)			0.0075*** (0.0410)	0.0050*** (0.0005)
CAR				0.0083*** (0.0011)				0.0048*** (0.0004)				0.0085*** (0.0011)
AR (1) (p-value)	0.0348	0.0351	0.0386	0.0388	0.0362	0.0362	0.0400	0.0397	0.0355	0.0358	0.0393	0.0394
AR (2) (p-value)	0.2876	0.2751	0.2911	0.2815	0.2898	0.2766	0.2959	0.2895	0.2871	0.2735	0.2914	0.2820
Sargan Test (p-value)	0.8501	0.8529	0.9316	0.8847	0.8509	0.8526	0.9097	0.9070	0.8522	0.8673	0.9279	0.8822
No. Of Cross-sections	42	42	42	42	42	42	42	42	42	42	42	42
No. Of Observations												

Notes: *** and ** denote the significance at the 1% and 5% levels, respectively. Figures in parentheses are standard errors.

**Table 8:** MG estimation.

Variable	Model 1a	Model 1 b	Model 1c	Model 1 d	Model 2a	Model 2 b	Model 2c	Model 2 d	Model 3a	Model 3 b	Model 3c	Model 3 d
Long-run coefficients												
CB Rate	−3.9855 (2.5549)	3.3797 (34.1594)	−0.1102 (1.5402)	20.4132 (22.8439)					2.1990 (4.0841)	1.2218 (1.4247)	−1.9951 (4.1397)	−3.4396 (3.9489)
MAPP					15.7212 (24.8159)	−16.3033 (19.8505)	41.5553** (17.4667)	−41.6036 (71.1352)	53.4928** (24.8410)	19.6299** (8.1844)	21.2314** (8.9004)	−9.4549 (23.4495)
INF	5.2130* (3.0207)	22.8610 (22.1759)	−0.6612 (1.1332)	−49.7433 (49.0934)	0.5476 (1.7173)	0.1144 (1.4985)	−0.3065 (0.5755)	4.6795* (2.7084)	0.0805 (2.3892)	−1.2613 (0.9930)	1.7736 (3.4784)	4.7368 (5.1306)
EG		−19.4921 (14.9440)	0.4697 (0.9614)	−2.1418 (4.8854)		−0.1460 (1.5981)	2.7596 (1.6863)	−7.4598 (5.3689)		0.3769 (0.6032)	1.0022 (0.9263)	−2.6531 (3.0287)
LDR			0.0762 (0.1118)	−15.7644 (15.2655)			−0.0553 (0.1414)	2.0215 (2.0419)			−0.1107 (0.0853)	−0.3321 (0.4619)
CAR				−12.0811 (12.3168)				2.0913** (1.0004)				−0.5638 (0.4550)
Short-run coefficients												
ECTt-1	−0.0038 (0.0113)	0.0260* (0.0146)	0.0499** (0.0208)	0.0250 (0.0260)	0.0019 (0.0097)	0.0162 (0.0180)	0.0505* (0.0299)	0.0365 (0.0274)	−0.0123 (0.0136)	0.0096 (0.0212)	0.0162 (0.0349)	−0.0262 (0.0339)
Δ(NPL (-1))	0.4582*** (0.0228)	0.4751*** (0.0238)	0.4809*** (0.0279)	0.4558*** (0.0252)	0.4804***							
(0.0147)	0.4853*** (0.0185)	0.4893*** (0.0267)	0.4565*** (0.0278)	0.4734*** (0.0181)	0.4704*** (0.0194)	0.4524 (0.0254)	0.4227*** (0.0265)					
Δ(CB Rate)	−0.1069 (0.0797)	−0.1541										
(0.0963)	0.1157 (0.1171)	0.0285 (0.2371)					−0.1493 (0.1019)	−0.1973 (0.1275)	−0.1170 (0.1785)	−0.0257 (0.3230)		
Δ(MAPP)					0.6735 (3.5165)	1.0670 (4.3115)	2.3974 (5.4735)	−2.3618 (7.3474)	0.2383 (3.6650)	−1.0078 (4.1436)	1.3626 (5.3415)	−7.6886 (6.6610)
Δ(INF)	−0.0523 (0.1036)	−0.1181 (0.1024)	−0.1538* (0.0869)	−0.1712* (0.0989)	−0.1122** (0.0441)	−0.1238** (0.0500)	−0.1256 (0.0585)	−0.1524 (0.1058)	−0.0192 (0.1135)	−0.0771 (0.1040)	−0.1181 (0.1032)	−0.2018 (0.1380)
Δ(EG)		0.0427 (0.0548)	0.1315 (0.0819)	0.0180 (0.1639)		−0.0055 (0.0501)	0.1318 (0.0902)	0.1821* (0.0982)		0.0125 (0.0582)	0.1072 (0.1047)	0.0227 (0.1733)
Δ(LDR)			0.0100 (0.0131)	−0.0922 (0.0649)			0.0065 (0.0162)	−0.0259 (0.0375)			0.0327** (0.0155)	−0.0548 (0.0721)
Δ(CAR)				0.0760 (0.0824)				0.0322 (0.0294)				0.0145 (0.0939)

Notes: *** and ** denote the significance at the 1% and 5% levels, respectively. Figures in parentheses are standard errors.

**Table 9:** Hausman test PMG - MG.

Variable	Model 1a	Model 1 b	Model 1c	Model 1 d	Model 2a	Model 2 b	Model 2c	Model 2 d	Model 3a	Model 3 b	Model 3c	Model 3 d
Chi^2^ (df)	1.06	1.39	3.48	5.65	2.95	3.04	0.48	0.76	2.14	0.76	4.40	4.64
*P* Value	0.5873	0.7068	0.4815	0.3420	0.2289	0.3859	0.9755	0.9443	0.5440	0.9437	0.4940	0.4613
Conclusion	PMG	PMG	PMG	PMG	PMG	PMG	PMG	PMG	PMG	PMG	PMG	PMG
No. Of Cross-sections	42	42	42	42	42	42	42	42	42	42	42	42

**Table 10:** DFE estimation.

Variable	Model 1a	Model 1 b	Model 1c	Model 1 d	Model 2a	Model 2 b	Model 2c	Model 2 d	Model 3a	Model 3 b	Model 3c	Model 3 d
Long-run coefficients												
CB Rate	−2.6933 (7.7934)	−1.5792 (6.1146)	−1.1955 (7.3689)	−0.3554 (5.6934)					−30.2443 (273.2324)	−41.8889 (425.8924)	−76.4005 (1541.666)	−30.3180 (280.6138)
MAPP					−329.7435 (2750.087)	−440.9159 (4468.231)	−877.5037 (18172.16)	−418.7025 (3714.19)	−440.7251 (4008.432)	−602.0141 (6158.142)	−1140.641 (23099.2)	−556.0032 (5162.995)
INF	−2.3644 (6.0616)	−2.4098 (5.8632)	−3.0734 (8.5106)	−2.0026 (5.4505)	−24.8473 (203.0634)	−23.0952 (230.3277)	−41.3211 (848.4482)	−16.4972 (143.9628)	−11.1555 (101.6902)	−5.9238 (64.4587)	−8.5452 (177.5329)	−6.2633 (21.2363)
EG		−0.8616 (2.9562)	−1.5918 (4.8270)	−2.3881 (5.1779)		−7.6467 (77.9642)	−19.7552 (408.1234)	−12.4398 (109.991)		−11.5890 (118.5771)	−26.1176 (527.8527)	−16.1689 (149.699)
LDR			−0.1398 (0.3459)	−0.1537 (0.3061)			−1.0091 (20.8157)	−0.6551 (5.7560)			−0.9615 (19.4257)	−0.6592 (6.0819)
CAR				0.2250 (0.4805)				1.3096 (11.4834)				1.2693 (11.6901)
Short-run coefficients												
ECTt-1	0.0048 (0.0097)	0.0053 (0.0097)	0.0041 (0.0097)	0.0051 (0.0097)	0.0012 (0.0099)	0.0009 (0.0099)	0.0004 (0.0099)	0.0011 (0.0099)	0.0011 (0.0099)	0.0009 (0.0099)	0.0004 (0.0099)	0.0010 (0.0099)
Δ(NPL (-1))	0.4973*** (0.0130)	0.4978*** (0.0130)	0.4977*** (0.0131)	0.4978*** (0.0131)	0.4957*** (0.0130)	0.4958*** (0.0130)	0.4959*** (0.0131)	0.4958*** (0.0131)	0.4956*** (0.0130)	0.4956*** (0.0130)	0.4957*** (0.0131)	0.4956*** (0.0132)
Δ(CB Rate)	−0.0897 (0.0750)	−0.1001 (0.0784)	−0.0914 (0.0784)	−0.0894 (0.0781)					−0.0115 (0.0790)	−0.1550* (0.0860)	−0.1414 (0.0859)	−0.1417* (0.0855)
Δ(MAPP)					0.7099 (3.0177)	1.4647 (3.3226)	2.0182 (3.3264)	1.1466 (3.3139)	1.1177 (3.1966)_	0.0851 (3.5699)	1.2173 (3.5762)	0.0936 (3.5627)
Δ(INF)	−0.0594 (0.0614)	−0.0729 (0.0636)	−0.0778 (0.0637)	−0.0707 (0.0634)	−0.1154*** (0.0424)	−0.0993** (0.0490)	−0.0943* (0.0490)	−0.0896 (0.0490)	−0.0366 (0.0729)	−0.0358 (0.0758)	−0.0357 (0.0757)	−0.0409 (0.0753)
Δ(EG)		−0.0087 (0.0413)	−0.0143 (0.0413)	−0.0147 (0.0421)		−0.0320 (0.0443)	−0.0393 (0.0443)	−0.0329 (0.0449)		−0.0217 (0.0447)	−0.0296 (0.0448)	−0.0234 (0.0453)
Δ(LDR)			−0.0027 (0.0021)	−0.0035 (0.0022)			−0.0021 (0.0021)	−0.0029 (0.0022)			−0.0020 (0.0021)	−0.0028 (0.0023)
Δ(CAR)				0.0105 (0.0062)				0.0108 (0.0062)				0.0105* (0.0063)

Notes: *** and ** denote the significance at the 1% and 5% levels, respectively. Figures in parentheses are standard errors.

**Table 11:** Hausman test PMG - DFE.

Variable	Model 1a	Model 1 b	Model 1c	Model 1 d	Model 2a	Model 2 b	Model 2c	Model 2 d	Model 3a	Model 3 b	Model 3c	Model 3 d
Chi^2^ (df)	4.42	1.56	4.76	0.88	1.51	2.39	3.75	7.34	1.82	2.53	4.61	7.80
*P* Value	0.1099	0.6678	0.3133	0.9720	0.4707	0.4962	0.4408	0.1969	0.6101	0.6400	0.4650	0.2532
Conclusion	PMG	PMG	PMG	PMG	PMG	PMG	PMG	PMG	PMG	PMG	PMG	PMG
No. Of Cross-sections	42	42	42	42	42	42	42	42	42	42	42	42

The long-term PMG Estimation results in Table 6 show that the central bank rate significantly and positively affected non-performing loans in the long term. Macro-prudential policy significantly and negatively affected non-performing loans in models 2 and 3. Moreover, inflation had a significant negative effect on non-performing loans in models 1 and 3. It had a significant negative effect on non-performing loans in model 2a but not in 2 b. Inflation also significantly and positively affected non-performing loans in 2 d, but not in 2c. Moreover, economic growth significantly and negatively affected non-performing loans in model 1 but had a significant positive effect in model 2. The effect of economic growth on non-performing loans was significant and negative in model 3 b, significant and positive in 3c, as well as positive and insignificant in 3 d. The findings also showed that the LDR significantly and negatively affected non-performing loans in models 1 and 2 but had a significant positive effect in model 3. Additionally, the CAR variable significantly and negatively affected non-performing loans in Models 1 d, 2 d, and 3 d.

The short-term PMG Estimation results showed that the central bank rate significantly and negatively affected non-performing loans in models 1, 2, and 3. The macro-prudential policy positively but insignificantly affected non-performing loans in models 2 and 3. Furthermore, the effect of inflation on non-performing loans was insignificant and positive in model 1. Its impact was significant and positive in models 2a and 2 d, insignificant and positive in 2 b, as well as insignificant and negative in 2c. Similarly, inflation negatively but insignificantly affected non-performing loans in models 3a and 3c, but had an insignificant positive effect in models 3 b and 3 d. The results also indicated that economic growth did not significantly and positively affect non-performing loans in the short term in all models. Similarly, the LDR and CAR had no significant positive effects on non-performing loans in the short term.

### 4.4 Robustness test

A robustness assessment was conducted to examine the impacts of the central bank rate and macro-prudential policy on the non-performing loans using Instrumental Variable (IV) estimates. This study employed [48] First Difference GMM (FD-GMM) estimator to account for potential endogeneity. In the first difference equation, the MPE was instrumented using lagged data because it was considered endogenous. Also, the control variables were considered exogenous because their levels were measured.

The Arellano-Bond FD-GMM estimation results in Table 7 show that the first-order autocorrelation AR (1) was significant. In contrast, the second-order autocorrelation AR (2) failed to reject the null hypothesis, meaning the FD-GMM estimation was consistent. The Sargan test was used to confirm the reliability of the instrumental variables. The results showed no correlation between the instruments and the residuals, confirming the Sargan test's null hypothesis. Therefore, the FD-GMM estimator was valid because the AR (1), AR (2), and Sargan tests showed that FD-GMM satisfied the panel IV model requirements.

The GMM Panel model estimation results showed that the central bank rate significantly and positively affected non-performing loans. Meanwhile, the macro-prudential policy and inflation had a significant negative effect on non-performing loans. Economic growth as well as LDR and CAR significantly and positively affected non-performing loans in all models.

**Figure: 1:**
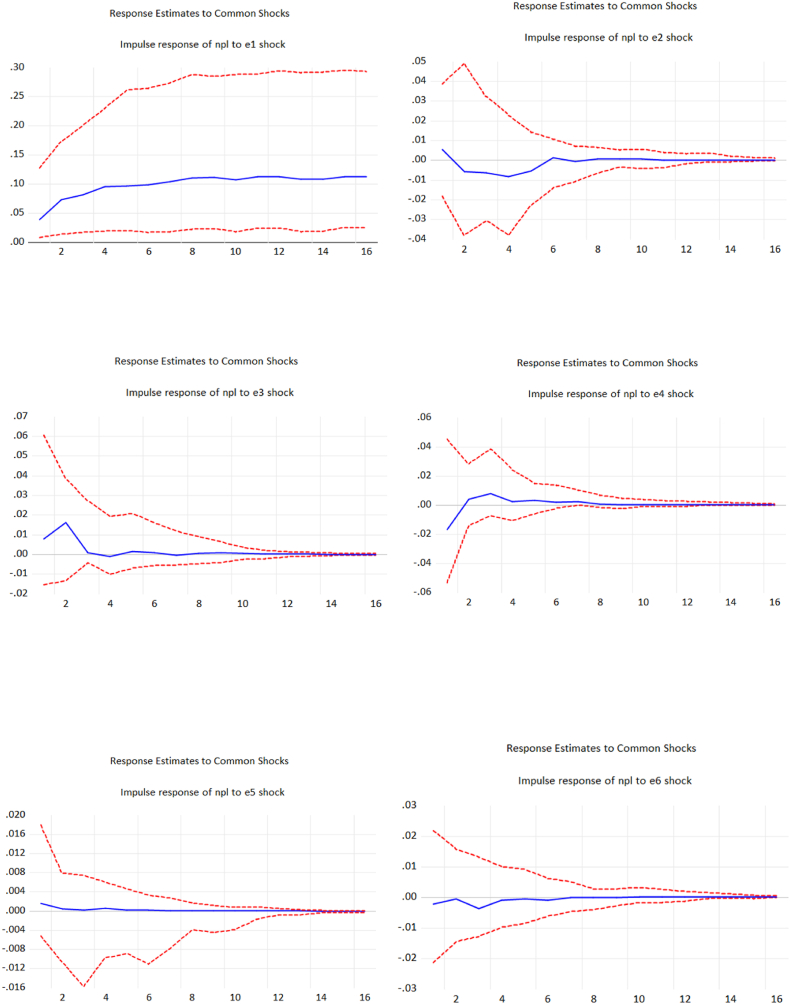
Impulse Response Function of Panel Structural VAR. Source: Author's computations.

Another way to solve endogeneity problem in the model is to perform Panel structural VAR developed by Ref. [49]. The results of Panel structural VAR is revealed in Fig. 1 in the appendix.

### 4.5 Discussion

The estimation results for the three models equations (2), (3), (4) using PMG are shown in Table 6. The findings indicate that monetary policy (CB rate) significantly and positively affects bank credit risk in the long term. This means that an increase in the CB rate or monetary policy tightening raises bank credit risk. The increase forces commercial banks to increase the lending rates charged to borrowers. Subsequently, the burden to be paid by the borrower increases, raising the possibility of default and non-performing loans [5,26]. found a significant positive effect of monetary policy through the central bank rate on credit risk. This finding supports [50] that higher credit interest charged to borrowers increases the possibility of bank credit failure.

The results showed that the CB rate significantly and negatively affects credit risk in the short term. In this case, the central bank's policy to lower the benchmark interest rate negatively affects non-performing loans. Loosening monetary policy by reducing interest rates leads to a large credit expansion and the possibility of banks disbursing loans to the public carelessly. This increases the ratio of non-performing loans to the possibility of defaulting on credit payments by borrowers, increasing banking risk. The results support [4,28,29] that monetary policy implemented by reducing interest rates encourages banks to make greater and less careful lending to the public, increasing risk.

Macro-prudential negatively and significantly affected bank credit risk in the long term in models 2 and 3. This means that tightening macro-prudential policies reduces bank credit risk. Tighter macroprudential policies make commercial banks more selective in extending credit, reducing the non-performing loans' credit risk. This finding supports [[31], [32], [33]] that macroprudential policy instruments have effectively mitigated credit growth. According to Refs. [19,51,52] tightening macro-prudential policies stabilizes the financial system. Meanwhile, the policy has positive results but an insignificant impact on credit risk in the short term.

The results showed that inflation negatively and significantly affects non-performing loans in the long run. This means that increasing inflation lowers the real value of loans and enables borrowers to repay loans on time, reducing the risk of credit failure. The results support [36,45,50] that inflation negatively affects non-performing loans. Meanwhile, inflation positively influences bank credit risk in short term. This is because higher inflation reduces company revenues and increases production costs, negatively impacting business actors and reducing profitability. The decline in profitability makes it difficult for companies to meet their loan obligations, increasing non-performing loans. These results support [29,35] that inflation positively influences non-performing loans.

Economic growth negatively and significantly affects non-performing loans in the long term. This means that an increase in the quality of a country's economy reduces the credit risk faced by banks. According to Ref. [29], a recession is characterized by an economic downturn, such as a sharp decline in consumer purchases. This reflects economic conditions that affect credit quality and increase bank risk. The results support [35,45] that economic growth is the borrowers' ability to fulfill their loan obligations to banks. Meanwhile, the positive and significant effect indicates that an increase in economic activity makes banks less careful in extending credit to the public and business actors, posing a large credit risk.

The LDR negatively and significantly affected non-performing loans in model 1. This indicated that an increase in LDR reduces bank credit risk. According to Ref. [45], a high LDR reduces the risk when the loans disbursed are more targeted. The results also showed that LDR significantly and positively affects bank credit risk. This means that an increase in LDR could be risky for bank credit quality, supporting [26,35,39,40]. Moreover, the CAR negatively and significantly affects non-performing loans in the long term. This means that the bank's capital represented by CAR must cover all business risks, including losses that occur due to non-performing loans. According to Refs. [[42], [43], [44], [45]], higher CAR increases the bank's ability to minimize credit risk. This reduces non-performing loans and increases the reserve funds obtained.

## 5 Conclusion

This study examined the influence of monetary and macro-prudential policies as well as other control variables on bank credit risk using the PMG approach in Indonesia. The results showed that the effect of monetary policy on credit risk is positive in the long run. This means that monetary policy tightening increases bank credit risk. On the contrary, the policy is expansive in the short term, reducing bank credit risk. Meanwhile, macro-prudential policy negatively affects credit risk. This finding implies that more prudent policies effectively reduce bank credit risk. Other results showed that macroeconomic and bank-specific variables exert more long-term rather than short-term effects. Therefore, better macroeconomic performance such as low and stable inflation, as well as higher economic growth are required to minimize non-performing loans in Indonesia's banking. The higher bank liquidity and capital also reduce credit risk in the long run.

The findings might help bank executives reconsider lending policies to minimize non-performing loans. The government must implement more effective macro-prudential and monetary policies to lower the ratio of non-performing loans in the Indonesian banking sector to maintain credit risk. Furthermore, this study made various contributions but had a few challenges. First, it used secondary data to assess the determinant factors of non-performing loans. Second, data were obtained from only 42 commercial banks operating in Indonesia as of the beginning of 2010. Therefore, future studies could incorporate other bank-specific parameters such as profitability and liquidity, as well as macroeconomic factors, including unemployment and exchange rate.

## Author contribution statement

Cep Jandi Anwar: Conceived and designed the experiments; Performed the experiments; Analyzed and interpreted the data; contributed reagents, materials, analysis tools or data; Wrote the paper.

Indra Suhendra: Conceived and designed the experiments; Analyzed and interpreted the data; Wrote the paper.

Eka Purwanda: Performed the experiments; contributed reagents, materials, analysis tools or data.

Agus Salim: Performed the experiments; contributed reagents, materials, analysis tools or data.

Nur Annisa Rakhmawati: Conceived and designed the experiments; Wrote the paper.

Ferry Jie: Conceived and designed the experiments; Analyzed and interpreted the data.

## Data availability statement

Data will be made available on request.

## Additional information

Supplementary content related to this article has been publish online at [URL].

## Declaration of competing interest

The authors declare that they have no known competing financial interests or personal relationships that could have appeared to influence the work reported in this paper
